# Aster-B Modulates Oxidative Stress Responses and Carotenoid Distribution in ARPE-19 Cells

**DOI:** 10.3390/antiox14050575

**Published:** 2025-05-10

**Authors:** Vidya Gopakumar, Johannes von Lintig

**Affiliations:** Department of Pharmacology, School of Medicine, Case Western Reserve University, Cleveland, OH 44106, USA; vxg255@case.edu

**Keywords:** retinal pigment epithelium, Aster-B, carotenoids, cholesterol, oxidative stress, CRISPR/dCas9

## Abstract

Lipid metabolism and oxidative stress are major contributors to ocular diseases, including drusen formation and photoreceptor damage. Aster-B, encoded by *GRAMD1B*, mediates the non-vesicular transport of cholesterol and carotenoids and is highly expressed in the human eye, though its specific ocular functions remain unknown. We investigated Aster-B’s role in ARPE-19 cells, a model of the retinal pigment epithelium (RPE), using CRISPR/dCas9 to generate an Aster-B-expressing cell line. Aster-B expression significantly improved cell survival under oxidative stress induced by hydrogen peroxide (H_2_O_2_) and was associated with the activation of the p53 and TGFβ signaling pathways, indicating a role in modulating stress responses. To confirm its lipid transport activity, we treated cholesterol-depleted cells with carotenoids and tracked their localization. In Aster-B-expressing cells, carotenoids accumulated in mitochondria, while in control cells, they remained in other cellular compartments. Under oxidative stress, mitochondrial carotenoid levels declined in Aster-B-expressing cells but not in control cells. Interestingly, carotenoids enhanced survival in control cells exposed to H_2_O_2_ but had a detrimental effect in Aster-B-expressing cells, suggesting that carotenoid function is context and location dependent. These findings highlight Aster-B’s role in coordinating lipid transport and stress responses in the RPE, with implications for oxidative stress-related eye diseases.

## 1. Introduction

The retinal pigment epithelium (RPE) lies between the neuronal retina and the choriocapillaris. It forms a crucial part of the outer retinal blood barrier. The epithelium is vital for ocular health due to performing functions such as the phagocytosis of photoreceptor outer segments, the maintenance of the visual cycle, and the regulation of the energy balance of the retina [1]. RPE cells are terminally differentiated and post-mitotic, a state that enables them to maintain their specialized roles in managing oxidative stress and retinal homeostasis over a lifetime but also renders them vulnerable to cumulative damage from reactive oxygen species.

The RPE also is a hub of ocular lipid metabolism and regulates the uptake of isoprenoid lipids, such as cholesterol, carotenoids, and retinoids, which are essential for photoreceptor function and survival [2,3,4]. Disruptions in lipid transport can lead to lipid accumulation at the basolateral membrane of the RPE or lipid deficiencies in photoreceptors, contributing to oxidative stress and inflammation [5]. This impairment is linked to ocular diseases such as age-related macular degeneration (AMD) and neurodegeneration [6,7]. Therefore, understanding lipid transport mechanisms at the RPE is critical for uncovering the molecular basis of associated diseases and identifying potential therapeutic targets.

Scavenger receptor class B type 1 (SR-B1) is expressed in the RPE and plays a crucial role in lipoprotein metabolism [8]. SR-B1 facilitates the selective uptake of lipids, including high-density lipoprotein (HDL)-derived cholesterol and dietary carotenoids such as lutein and zeaxanthin [9,10], by loading them into the plasma membrane (PM). Recent studies have revealed that Aster proteins, encoded by the *GRAMD1A*, *B*, and *C* genes, operate downstream of SR-B1, aiding in the transportation of lipids through non-vesicular transport mechanisms [11,12].

Aster proteins exhibit a tripartite structure featuring a StART-like lipid-binding domain flanked by an N-terminal GRAM domain and a C-terminal membrane anchor [12]. The crystal structure of the lipid-binding domain of Aster-A, with bound cholesterol, has been solved [12], and subsequent studies have demonstrated its capability to accommodate rigid C40 carotenoids [13,14]. Binding assays with recombinant Aster-A and B have confirmed that carotenoids can compete with cholesterol for binding to their StART-like domains [14], indicating that Aster proteins display transport activity with several lipid classes. Aster proteins form contact points between the endoplasmic reticulum (ER) and PM, as well as between the ER and mitochondria [15], thereby facilitating the non-vesicular transport of cholesterol and carotenoids between different cellular compartments. *GRAMD1A-C* genes are expressed in several cell types and organs [16]. A critical role of *GRAMD1B*, encoding the Aster-B protein, has been established in cholesterol absorption in the enterocytes of the small intestine [17]. Moreover, Aster-B plays a critical role in steroidogenesis in the adrenal glands [12]. The *GRAMD1B* gene is also highly expressed in several cell types of the human retina and the RPE [14]. However, the ocular role of Aster-B has not been studied in detail.

To investigate the role of Aster-B in ocular lipid metabolism, we utilized ARPE-19 cells, a human-derived cell line that retains many characteristics of primary RPE cells and is widely used in ocular disease research. Since ARPE-19 cells lack endogenous expression of the *GRAMD1B* gene [13], we employed CRISPR/dCas9 gene activation to induce its expression. To mimic the disease states of the RPE, we exposed cells to oxidative stress induced by hydrogen peroxide. Our study demonstrated that Aster-B facilitated lipid transport in ARPE-19 cells and influenced the cellular response to oxidative stress. These results uncover a novel role for Aster-B in oxidative stress defense, linked to lipid metabolism in ocular cells.

## 2. Materials and Methods

### 2.1. Reagents

H_2_O_2_, DCFH-DA, anti-COX IV antibody (Sigma, St. Loius, MO, USA), CyQUANT MTT Cell Viability Assay kit (V131540, Thermo Fisher, Waltham, MA, USA),TF Activation Profiling Plate Array (FA-1005) and Nuclear Extraction kit (SK-001) (Signosis, Santa Cruz, CA, USA). Control CRISPR Activation Plasmid (sc-437275), *GRAMD1B* CRISPR Activation Plasmid (h) (sc-408929-ACT), plasmid transfection medium (sc-108062), Ultra Cruz transfection reagent (sc-395739), hygromycin B (sc-29067), puromycin (sc-108071), and blasticidin S HCl (sc-204655A) (Santa Cruz Biotechnology, Dallas, TX, USA). Anti-*GRAMD1B* Polyclonal antibody (24905-1-AP, Proteintech, Rosemont, IL, USA), anti-Caspase-3 #9662, anti-NRF2 #20733S, anti-HO-1 #43966S, anti-P53 #9282 (Cell Signaling Technology, Danvers, MA, USA), Alexa Fluor 488-conjugated goat anti-rabbit IgG (A11008) HRP-conjugated goat anti-mouse and anti-rabbit IgG (H + L) (Invitrogen, Carlsbad, CA, USA), DAPI mounting medium (Southern biotech, Birmingham, AL, USA), HRP-conjugated secondary antibodies (Abcam, Waltham, MA, USA), Mitochondrial isolation kit, M-Per, Protease Inhibitor, Pierce ECL Western blotting substrate, BCA assay kit (Thermo Scientific, Waltham, MA, USA), PDVF membrane (Bio-Rad, Hercules, CA, USA), TaqMan primers (Thermo fisher, Waltham, MA, USA), TaqMan fast universal PCR master mix (Applied Biosystems, Foster City, CA, USA).

### 2.2. Cell Culture

The human ARPE-19 cell line was obtained from the American Type Culture Collection (ATCC), USA. ARPE-19 *GRAMD1B*-null ARPE-19 (−) and *GRAMD1B*-stable ARPE-19 (+) cells were cultured in DMEM/F-12 (1:1) medium (Gibco), supplemented with 2.5 mM L-glutamine, 15 mM HEPES buffer, 10% fetal bovine serum (FBS) (Gibco), and 1% (*v*/*v*) antibiotic antimycotic (Gibco) at 37 °C and 5% CO_2_. Typically, cells were seeded in a 24-well plate, a 6-well plate, or a 10 cm dish at a concentration of 150 K and 400 K per well and 2 × 10^7^ cells per dish and grown overnight at 37 °C.

### 2.3. CRISPR/dCas9 Activation of Endogenous GRAMD1B Gene Expression

Transfection with plasmid DNA and the selection of *GRAMD1B*-stable clones were performed according to the manufacturer’s instructions. Initially, the optimal plasmid DNA transfection reagent ratio (manufacturer) was determined experimentally and optimized to minimize cell toxicity. For this purpose, the appropriate ratio of plasmid DNA to transfection reagent (manufacturer) used per well was established to determine which amount provided the highest transfection efficiency. The following solutions were prepared. Solution A: For each transfection, 0.5–2 µg of plasmid DNA and plasmid transfection medium were mixed to a final volume of 150 µL. The mix was incubated for 5 min at room temperature. Solution B: For each transfection, 5–15 µL of Ultra Cruz transfection reagent was diluted with plasmid transfection medium to a final volume of 150 µL and incubated for 5 min at room temperature. Then, solution A was added dropwise to solution B using a pipette. The combined solutions were vortexed immediately and incubated for 20 min at room temperature. Prior to transfection, the culture medium was replaced with fresh antibiotic-free growth medium, and a 300 µL plasmid transfection medium (combined solutions A and B) was added dropwise to the cells and gently mixed by swirling the plate.

For clonal selection, the human ARPE-19 cells seeded in a 6-well plate at a 40 to 60% confluency were transfected with scrambled plasmid DNA or *GRAMD1B* plasmid DNA at a concentration of 1.5 (µg/well) and cultured in normal medium for 60 h at 37 °C. The normal medium was replaced with 3 mL of the selection medium containing all hygromycin, blasticidin, and puromycin at concentrations of 200, 7.5, and 7.5 µg/mL of medium, respectively, following two rounds of antibiotic selection for a total duration of five days. The antibiotic-resistant scrambled and *GRAMD1B* stable clones were pooled and expanded in the normal medium for five days. Cells were then immediately used for further studies or frozen in liquid nitrogen for storage until future use.

### 2.4. Staining for Reactive Oxygen Species

ROS levels were determined using a fluorescent probe, 2,7-dichlorofluorescin diacetate (DCFH-DA, Sigma). Briefly, ARPE-19 cells were seeded at a density of 2 × 10^5^ cells per well in a 24-well plate and grown overnight at 37 °C. Then, cells were treated, respectively, with a defined dose of hydrogen peroxide for 24 h. Untreated cells served as controls. After washing cells with phosphate-buffer saline (PBS), medium containing 10 μM of DCF-DA was added for 30 min at 37 °C. After treatment, cells were washed once with DMEM and twice with PBS. After washing, 500 μL of PBS was added to each well, and the staining of the cells was observed and photographed using the green fluorescent protein (GFP) channel of a fluorescence microscope (Leica DMI6000B, Leica Microsystems, Wetzlar, Germany) equipped with a digital camera.

### 2.5. Treatment of Cells with Carotenoids and Hydrogen Peroxide

ARPE-19 cell lines were seeded in a 6-well plate or in a 10 cm dish at a concentration of 300 K and 2 × 10^7^ cells/well or dish and grown until they reached ~90% confluency. Then, the medium was changed to a serum-free medium with 2 mM methyl-β-cyclodextrin (MCD) (Thermo Scientific) for 2 h at 37 °C in 5% CO_2_. Serum-free medium was then mixed with 2 mM MCD and 2 μM of zeaxanthin dissolved in acetone (<1% (*v*/*v*) final concentration). The cells were incubated for 24 h with the zeaxanthin/MCD mix to achieve cellular carotenoid uptake and transport. Cells were then washed and incubated in the presence of various concentrations of H_2_O_2_ for 24 h. Cells were then washed with PBS three consecutive times before they were collected by scraping and centrifugation. The collected cell pellets were either immediately subject to analyses or stored at −80 °C until further use.

### 2.6. Total Cell Lysate, Mitochondrial, Cytosolic, and Nuclear Extract Isolation

The total, mitochondrial, cytosolic, and nuclear fractions of the ARPE-19 cells were prepared according to the manufacturer’s instructions using the M-PER (Thermo Scientific), mitochondrial (Thermo Scientific), and Nuclear Extraction (Signosis) kits, respectively. For the isolation of total cell lysates, cell pellets were suspended in a 200 to 400 μL M-PER buffer containing a Protease Inhibitor cocktail (Thermo Scientific) and homogenized gently for 5 min. The samples were centrifuged at 15,000 rpm for 15 min to pellet the cell debris. The supernatant containing total cell lysates was used for further studies. For the isolation of mitochondrial and cytosolic fractions, 800 μL of mitochondria isolation reagent A was added to the cell pellets, vortexed for 5 s at medium speed and incubated on ice for two minutes. Then, 10 μL of mitochondria isolation reagent B was added, and the mix was vortexed at maximum speed for 5 s. The mix was incubated on ice for 5 min, and vortexing at maximum speed was repeated every minute. Then, 800 μL of mitochondria isolation reagent C was added, and the tubes were inverted several times to mix the solution. The extracts were then centrifuged at 700× *g* for 10 min at 4 °C. The pellet was discarded, and the supernatant was centrifuged at 12,000× *g* for 15 min at 4 °C. The supernatant containing the cytosolic fraction was saved, and the pellet containing the mitochondria was washed once with 500 μL of mitochondria isolation reagent C and centrifuged at 12,000× *g* for 5 min. For the isolation of nuclear extracts, 250 μL of buffer 1 was added to the cell pellet (1 × 10^6^ cells/well), and the tubes were rocked at 200 rpm for 10 min on a shaking platform. The tubes were centrifuged at 12,000 rpm for 5 min at 4 °C, and the supernatant was discarded thoroughly and completely. In total, 50 μL of freshly prepared Buffer II was added to the pellet, and this allowed the pellet to gently float in the Buffer II working reagents. The tubes were placed on ice and shaken at 200 rpm for two hours. Then, the samples were centrifuged at 12,000 rpm for 5 min at 4 °C. The supernatants containing the nuclear extract were transferred to a new tube and stored on ice until further analysis. A BCA assay (Thermo scientific) was used to determine the protein concentration of the different subcellular fractions. Typically, the protein concentrations in different cellular fractions ranged as follows: total cell lysates (15 to 20 µg/µL); mitochondria (8 to 10 µg/µL); cytosol (1 to 2.5 µg/µL); nuclear extracts (2 to 3 µg/µL).

### 2.7. SDS-PAGE and Immunoblotting

Total soluble protein lysates from ARPE19-null and -stable cell pellets treated as described above were extracted using M-PER (Mammalian Protein Extraction Reagent) (Thermo scientific), containing Protease Inhibitor (Thermo scientific). The protein concentration was determined by the BCA (Thermo scientific) assays. In total, 30 to 60 μg of protein per lane was denatured in 2 × gel-loading buffer (4% SDS, 20% glycerol, 120 mM Tris-HCl (pH 6.8), and 0.01% bromophenol blue, with 10%‚ β-mercaptoethanol), heated to 95 °C for 5 min, and resolved by SDS-PAGE. Proteins were transferred to the PVDF membrane for 2 h and blocked for one hour at room temperature. The blots were incubated overnight at 4 °C with the primary antibody with 5% BSA in TBST. The antibodies used included *GRAMD1B* (Proteintech), COXIV, Caspase-3, PARP, and Histone H3 (Cell Signaling Technology) at a dilution of 1:1000. For protein loading controls, anti-β-actin (Cell Signaling) was used at a dilution of 1:2000. The blots were washed with TBST and incubated with secondary anti-rabbit IgG antibody or anti-mouse IgG antibody (Abcam) at a dilution of 1:5000 in 5% milk in TBST for one hour at room temperature. The blots were washed with 1X TBST thrice. The PierceTM ECL Western blotting substrate (Thermo scientific) was added to the blots for 1–3 min and was scanned with the Odyssey Imaging System (LI-COR Biosciences, Lincoln, NE, USA) for chemiluminescence detection.

### 2.8. Carotenoid Extraction and HPLC Analysis

Zeaxanthin (β,β-carotene-3R,3R’-diol) was a gift from DSM (Sisseln, Switzerland). Zeaxanthin was extracted from total cell lysate, mitochondrial, and cytoplasm fractions, using previously published protocols [13], and subjected to HPLC analysis on a silica column using hexanes: ethyl acetate (70:30 *v*/*v*) as the mobile phase. In a typical zeaxanthin extraction, 200 µL of total cell lysate, 100 µL of the mitochondrial fraction, and 750 µL of cytosolic fractions were mixed with the same amount of methanol. Then, 400 µL of acetone, 400 µL of diethyl ether, and 100 µL of petroleum ether were added. The mixture was vortexed two times for 15 s. Phase separation was achieved by centrifugation for one minute at room temperature (2000× *g*). The organic phase was transferred to a new tube and vacuum-dried using a speed vacufuge (Eppendorf). The extracted zeaxanthin was reconstituted in 150 uL of hexane/ethyl acetate (70:30 *v*/*v*) solvent. HPLC analysis was carried out with an Agilent 1260 Infinity Quaternary HPLC system (Santa Clara, CA, USA) equipped with a pump (G1312C), with an integrated degasser (G1322A), a thermostated column compartment (G1316A), an autosampler (G1329B), a diode array detector (G1315D), and online analysis software (Agilent OpenLab CDS Chemstation, A.01.02). The analyses were carried out at 25 °C using a normal-phase Zorbax Sil (5 μm, 4.6 × 150 mm) column (Agilent Technologies, Santa Clara, CA, USA). Chromatographic separation was achieved by isocratic flow at 1.4 mL per minute. Zeaxanthin and its metabolic products were identified based on the absorption spectrum of their peaks. The integration area for each peak was calculated, and the picomole amount of zeaxanthin was determined based on scaling the column with known amounts of zeaxanthin standard and normalized to the mg protein amount of the samples.

### 2.9. Oxidative Stress Transcription Factor Activation Profiling

The oxidative stress transcription factor activation profiling assay (Signosis) was used for monitoring the activation of oxidative stress-related transcription factors (TFs) following the manufacturer’s instructions. Briefly, the nuclear extracts of ARPE-19 cells were mixed with a series of biotin-labeled DNA probes based on the consensus sequences of transcription factor DNA-binding sites and incubated at RT for 30 min. After the incubation, the bound TFs were separated from the free TFs through spin column purification. Next, the bound probe was eluted and analyzed through hybridization with a hybridization plate where each well was precoated with complementary DNA sequences of the TF probe. The assay allowed the simultaneous analysis of the activation of 16 oxidative stress-related TFs. In total, 100 µL of denatured probes were diluted in 10 mL of warmed hybridization buffer, and 95 µL of the mixture was added into the corresponding 16 wells, and the plate was incubated at 42 °C overnight. The wells were washed thrice with the hybridization wash buffer and blocked for 15 min with the blocking buffer. After 15 min of blocking, 20 μL of Streptavidin-HRP Conjugate in 10 mL of blocking buffer (1:500 dilution) was added to each well and incubated for 45 min at room temperature with gentle shaking. After 45 min, a 200 μL detection wash buffer was added to each well, and the plate was incubated for 5 min with gentle shaking on a plate-shaker at room temperature. The wells were washed thrice for a total of 15 min. After the last wash, 1 mL of substrate A and 1 mL of substrate B were mixed with 8 mL of substrate dilution buffer and 95 μL of substrate solution in each well and incubated for 1 min. The captured DNA probe was detected with the diluted Streptavidin-HRP Conjugate. The plate was placed in the microplate luminometer and read within 5 min. Luminescence is reported as relative light units (RLUs).

### 2.10. Cell Viability Assays

Cell viability was measured by an MTT assay (CyQUANT MTT, Thermo scientific). Briefly, ARPE-19 cells were seeded in 96 96-well plates at a density of 4 × 10^3^ cells per well and grown overnight at 37 °C. Cells were left either untreated or treated with H_2_O_2_ in the presence or absence of zeaxanthin for 24 h in serum-free medium. Following treatment, the cells were washed with PBS 3 times and were incubated with a 10 μL MTT solution for 4 h. This was followed by 100 μL of the SDS-HCL solution for 4 h at 37 °C. The amount of formazan dye was measured by detecting the absorbance at a wavelength of 570 nm with a microplate reader (Versamax Microplate Reader, Molecular devices). Eight readings per sample were taken to reduce random errors, and the average was calculated.

### 2.11. qRT-PCR Analysis of Marker Genes

Total RNA was isolated from ARPE19 cells using TRIZOL reagent (Invitrogen, Carlsbad, CA, USA) and the RNeasy Mini Kit (Qiagen, Germantown, MD, USA). RNA concentration and purity were determined with a nano-drop spectrophotometer (Thermo Scientific). Isolated RNA was converted to cDNA using the High-Capacity Reverse Transcription Kit (Applied Biosystems, Foster City, CA, USA). Gene expression analysis was carried out by real-time quantitative PCR using an Applied Biosystems real-time PCR instrument with Taq Man probes (Thermo Fisher Scientific). Primers used for analysis were human *GAPDH* (Hs99999905) and *BCO2* (Hs01568558). We used 20 ng of cDNA in a 10 μL reaction, and the amplification was carried out using TaqMan polymerase Fast Universal PCR Master Mix (2×) No Amp Erase, UNG (Applied Biosystems), following the manufacturer’s protocol. Gene expression levels were normalized to the expression of the housekeeping gene GAPDH using the ∆∆Ct method.

### 2.12. Immunocytochemistry and Confocal Imaging

ARPE19 (−) and ARPE19 (+) cells were grown on Labtek chamber slides (Nunc Thermofisher, Walthamn, MA, USA) for 24 h at 37 °C in a 5% CO_2_ incubator. Cells were washed with 1 × phosphate-buffered saline (PBS) and fixed with 4% paraformaldehyde (Electron Microscopy Sciences, Hatfield, PA, USA) for 15 min at room temperature. Cells were washed and permeabilized with 0.2% Triton X-100 (Sigma) for 15 min. Cells were washed and blocked with 5% goat serum in PBST (Tween 20, 0.05%) for 1 h at 37 °C and incubated with primary antibodies GRAMD-1B (2 μg/mL), NRF2, p53, and HO-1 (0.4 μg/mL) at 4 °C overnight. After a wash with PBST buffer, cells were incubated with secondary antibody Alexa Fluor rabbit 488 and Alexa Fluor mouse 555 (4 μg/mL) at room temperature for 2 h. DAPI Fluoromount G (Southern biotech) was used to stain the nucleus. Images were taken with an Olympus FV1200 laser scanning confocal microscope (Olympus America, Waltham, MA, USA) using a 405 nm diode laser for the blue channel, 473 nm diode laser for the green channel with a UPLXAPO100XO oil OFN26.5, NA1.45 objective.

### 2.13. Statistical Analysis

Data shown are the mean ± SD and are representative of more than two or three independent experiments. Analysis was performed using an unpaired two-tailed *t* test and one-way ANOVA using GraphPad Prism 10.2 software, and results are considered significant at * *p* < 0.05, ** *p* < 0.005, and **** *p* < 0.0001.

## 3. Results

### 3.1. Generation of an Aster-B-Expressing ARPE-19 Cell Line

We aimed to explore Aster-B’s role in ARPE-19 cells, a widely used in vitro model for the RPE [18]. However, our previous study revealed that ARPE-19 cells do not express the *GRAMD1B* gene [13]. To overcome this limitation, we employed CRISPR/dCas9-mediated genome editing to induce *GRAMD1B* expression in ARPE-19 cells. For this purpose, we utilized synergistic activation mediator (SAM) CRISPR/dCas9 Activation Plasmids, specifically designed to upregulate human *GRAMD1B* (Figure S1A). ARPE-19 cells were transfected with these plasmids alongside scrambled control DNA as a negative control. Post-transfection, we selected stable *GRAMD1B*-expressing and control cell populations by culturing them in a medium containing selective antibiotics for five days. The antibiotic-resistant colonies were then expanded in standard medium for an additional week (Figure S1B,C).

**Figure 1:**
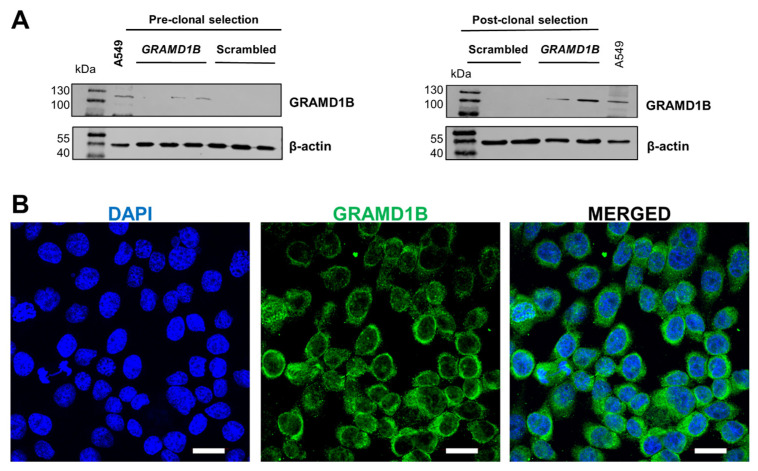
CRISPR/dCas9 activation of endogenous *GRAMD1B* gene expression in ARPE19 *GRAMD1B*-null cells. (**A**) ARPE-19 cells were transfected with scrambled DNA at 0.5, 1, and 1.5 μg DNA per well and *GRAMD1B* plasmid DNA at 0.5, 1, and 1.5 μg DNA per well, respectively. Western blot analysis was conducted with 50 μg of total protein per lane and showed that *GRAMD1B* is expressed before clonal selection and becomes enriched in post-clonal ARPE-19 cells (25 and 50 μg per well) treated with *GRAMD1B* plasmid DNA (1.5 μg per well). In contrast, *GRAMD1B* expression is absent in control cells treated with scrambled control DNA (1.5 μg per well). A549 cells were used as a positive control for *GRAMD1B* expression. β-Actin was used as a loading control. (**B**) Confocal imaging analysis with ARPE-19 cells transfected with plasmid DNA confirmed that *GRAMD1B* protein is expressed and mostly localized in the cytoplasm. Similar results were obtained from WB and ICC in more than three independent experiments. The scale bar is 10 μm.

Following expansion, cells were harvested, and protein was isolated, or total cell lysates were prepared. To confirm the successful activation of *GRAMD1B* expression in ARPE-19 cells, we performed Western blot analysis. The antibody detected a major band of 110 kDa in the plasmid DNA-treated cells, which was absent in the control cells treated with scrambled DNA (Figure 1A). This size of the band corresponded to the Aster-B protein observed in A549 cells (Figure 1A), a cell line known to express Aster-B [13].

To further validate Aster-B expression, we performed immunocytochemistry on the stable ARPE-19 cells. The confocal imaging of stained cells revealed Aster-B localization around the nuclei, as indicated by DAPI staining (Figure 1B). The staining was distributed throughout the cytoplasm, consistent with our previous reports on this protein [13]. Thus, we successfully established Aster-B-expressing ARPE-19 (+) cells and generated corresponding ARPE-19 (−) control cells, providing a robust platform for our further analysis.

### 3.2. Aster-B Modulates Responses to Oxidative Stress

**Figure 2:**
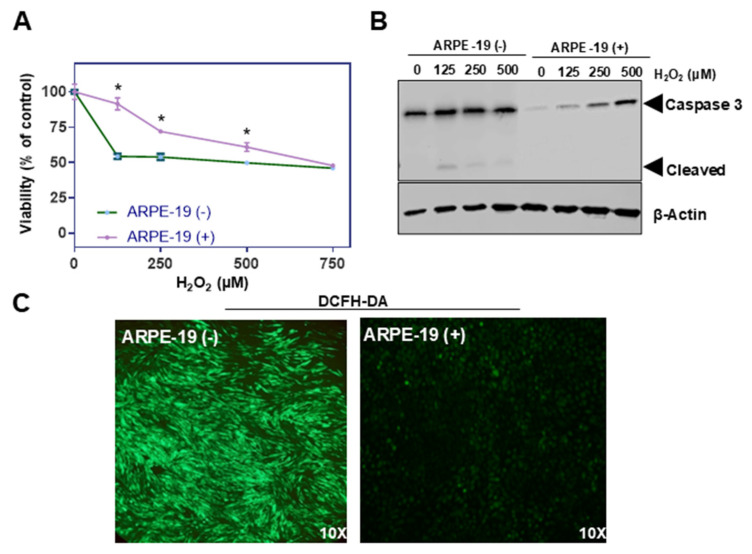
Aster-B expression improves resistance to H_2_O_2_-induced cell death. (**A**) Sensitivity of ARPE-19 (+) and ARPE-19 (−) cells toward H_2_O_2_-induced cell death. (**B**) Western blot of untreated and 0.125, 0.25, and 0.5 mM of H_2_O_2_ treated for 24 h in ARPE-19 (−) and ARPE-19 (+) cell lysates probed with anti-Caspase-3 and β-actin as a loading control induced a dose dependent cleavage of capase-3 with a concomitant generation of cleaved caspase-3, suggesting that H_2_O_2_ induces ARPE19 cell death via ferroptosis. (**C**) Representative images of ARPE-19 (−) and ARPE-19 (+) cells stained with DCFH-DA. Cells were treated with 0.25 mM H_2_O_2_ for 24 h prior to staining. Similar results were obtained from viability, WB, and ROS staining in three independent experiments. Analysis was performed using an unpaired two-tailed *t* test using GraphPad Prism 10.2 software, and results are considered significant at * *p* < 0.05.

Oxidative stress is a key factor implicated in the pathogenesis of various ocular disorders, including AMD [7]. The susceptibility of ARPE-19 cells to oxidative damage makes the cell line a versatile in vitro system for exploring the underlying pathologies [19]. To evaluate the putative role of Aster-B protein in this process, we determined the survival rates of ARPE-19 (+) compared to ARPE-19 (−) control cells when exposed to increasing concentrations of hydrogen peroxide (H_2_O_2_). Thus, we treated ARPE-19 (+) and ARPE-19 (−) cells with increasing concentrations of hydrogen peroxide (0, 125, 250, 500, and 750 µM) for 24 h to induce oxidative stress. Cell viability was measured post-treatment using MTT (3-(4,5-dimethylthiazol-2-yl)-2,5-diphenyltetrazolium bromide) assays [20]. Results showed that ARPE-19 (+) cells had higher survival rates, particularly at lower concentrations of H_2_O_2_ compared to ARPE-19 (−) cells (Figure 2A).

Oxidative stress disrupts mitochondrial function, and this damage promotes the release of cytochrome c, which further activates the caspase cascade, leading to apoptosis. This pathway is central to oxidative damage in RPE cells and is implicated in degenerative conditions like AMD. Caspase-3 plays a crucial role in the mitochondrial apoptotic pathway of human retinal pigment epithelial cells, including the ARPE-19 cell line [21,22]. Therefore, we determined pro-caspase and cleaved caspase-3 levels in ARPE-19 (+) and ARPE-19 (−) cells by Western blot analysis. We observed significant amounts of cleaved caspase-3 in ARPE-19 (−) cells treated with hydrogen peroxide (Figure 2B). Interestingly, pro-caspase-3 but not cleaved caspase-3 levels increased in ARPE-19 (+) cells when exposed to the same treatment regimen (Figure 2B).

To directly measure intracellular ROS levels upon treatment, cells were stained with 2′,7′-dichlorodihydrofluorescein diacetate (DCFH-DA), a fluorescent probe that detects ROS by emitting green fluorescence upon oxidation. Fluorescence microscopy was used to visualize the green fluorescence intensity. *GRAMD1B*-expressing cells showed significantly less green fluorescence than control cells treated with hydrogen peroxide, suggesting lower ROS accumulation in the presence of the Aster-B protein (Figure 2C).

### 3.3. Aster-B Modulates the Cellular Responses of ARPE-19 Cells to Oxidative Stress

**Figure 3:**
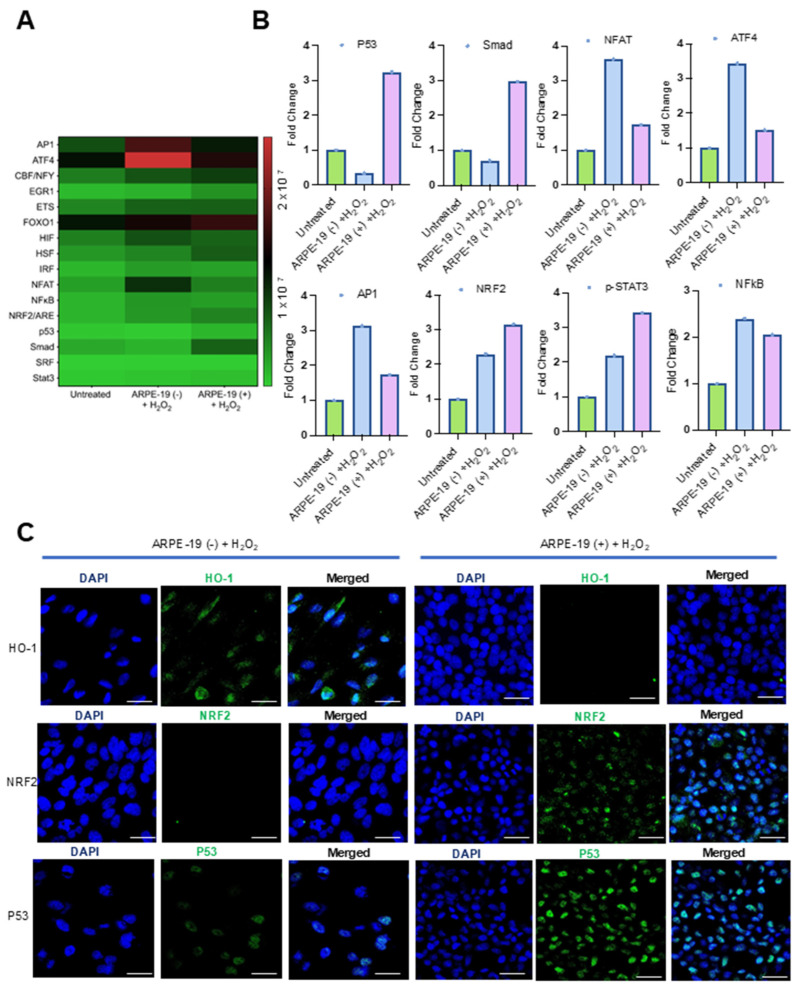
Effects of Aster-B expression on the DNA-binding activity of oxidative stress-relevant transcription factors. (**A**) Heatmap displays the expression profiles of 16 TFs in ARPE-19 (−) and ARPE-19 (+) cells treated as indicated in the *Y*-axis of the figure. (**B**) Fold changes in expression levels of oxidative stress-relevant transcription factors in ARPE-19 (+) cells compared to ARPE-19 (−) cells treated with 0.25 mM H_2_O_2_ alone. (**C**) Representative confocal imaging analysis from two independent experiments for HO-1, NRF2, and p53 expression levels in ARPE-19 (−) and ARPE-19 (+) cells treated with 0.25 mM H_2_O_2_ alone for 24 h. Nuclei are stained with DAPI. The scale bar is 10 μm.

We observed that *GRAMD1B* expression improved the survival of ARPE-19 cells when exposed to oxidative stress. To understand the putative mechanisms underlying this effect, we explored oxidative stress-responsive signaling pathways. These pathways control the transcription of genes encoding antioxidant enzymes and DNA repair enzymes that counteract oxidative damage in cells. To measure the activation of these signaling pathways, we took advantage of a commercial oxidative stress transcription factor (TF) activation profiling array. The array allows the simultaneous monitoring of 16 of the most common consensus DNA-binding sites of oxidative stress-activated TFs (Figure 3A). To assess the activation of these TFs, we isolated nuclear extracts and conducted assays following the protocol described in the Materials and Methods Section. Hydrogen peroxide treatment led to the enrichment of several TFs at their consensus DNA-binding sites when compared to untreated cells (Figure 3A,B). In ARPE-19 (+) cells, p53 and Smad/TGFβ were specifically enriched in nuclear extracts (Figure 3B). In ARPE-19 (−) cells, the nuclear factor of activated T cells (NFAT), activating transcription factor 4 (ATF4), and activator protein 1 (AP-1) were the most enriched TFs upon H_2_O_2_ treatment (Figure 3B). In both cell lines, nuclear factor erythroid 2-related factor 2 (NRF2), p-STAT3, and NFκB were increased over control cell levels (Figure 3A,B).

To validate these findings, we performed immunocytochemistry for p53 and NRF2 (Figure 3C). Strong signals for p53 became detectable in the nuclei of H_2_O_2_-treated ARPE-19 (+) cells, whereas the signal was largely absent in ARPE-19 (−) cells (Figure 3C). Also, the nuclear staining of NRF2 was stronger in ARPE-19 (+) cells, and the staining of the NRF2 target heme-oxygenase-1 was stronger in ARPE-19 (−) cells (Figure 3C). We also performed Western blot analysis for HO-1 and confirmed these results (Figure S2).

### 3.4. Activated GRAMD1B Facilitates Carotenoid Transport to Mitochondria

**Figure 4:**
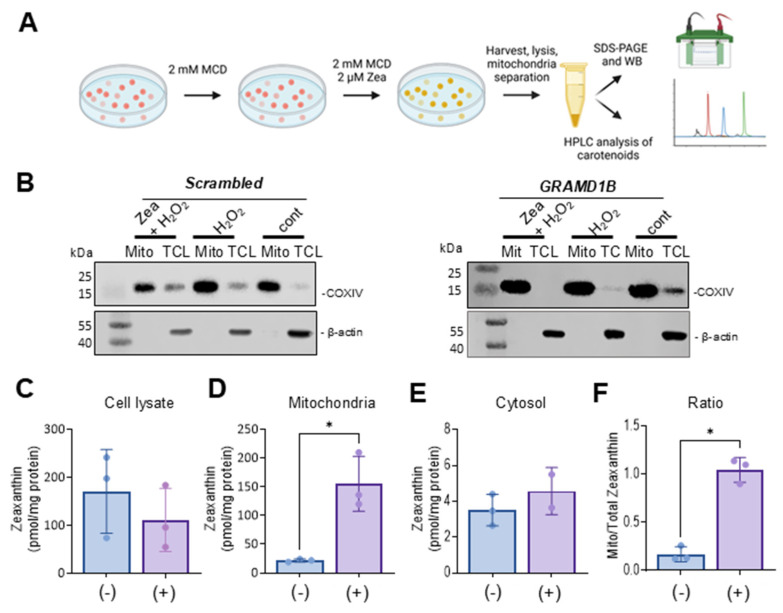
Aster-B expression stimulates mitochondrial uptake of zeaxanthin. (**A**) Scheme of the experimental procedure. ARPE-19 (+) and ARPE-19 (−) were treated with 2 mM MCD to mildly deplete the plasma membrane of cholesterol. Then, cells were incubated in the presence of 2 mM MCD and 2 μM of zeaxanthin. Cells were harvested, lysed, and separated into different compartments of SDS-PAGE and HPLC analyses. (**B**) Western blot of ARPE-19 (+) and ARPE-19 (−) cells: mitochondrial fraction and total cell lysate probed with anti-actin and anti-COXIV antibodies. (**C**) Zeaxanthin concentration of total cell lysate, (**D**) mitochondria, (**E**) cytosol, and (**F**) ratio of mitochondrial to cytoplasm zeaxanthin concentration in ARPE-19 (+) and ARPE-19 (−) cells. Similar results were obtained from WB and HPLC analysis in more than two independent experiments. The data shown are the mean ± SD. Analysis was performed using an unpaired two-tailed *t* test using GraphPad Prism 10.2 software, and results are considered significant at * *p* < 0.05.

We assumed that the protective effects of Aster-B on ARPE-19 cells were related to its function in moving lipids, i.e., sterols, between cellular membranes. To directly test whether the activation of *GRAMD1B* gene expression resulted in the movement of lipids, we took advantage of carotenoids that can be easily distinguished from endogenous lipids by their spectral properties [13]. Thus, we treated ARPE-19 (+) cells with 2 mM methyl-β-cyclodextrin (MCD) to mildly deplete endogenous cholesterol from the PM. After this pretreatment, cells were incubated in the presence of 2 mM MCD and 2 µM of zeaxanthin to load the PM with carotenoids. After 24 h, cells were harvested and fractionated into cytoplasmic and mitochondrial components (Figure 4A). ARPE-19 (−) cells underwent the same procedure and served as controls in these experiments.

The purity of the mitochondrial and cytoplasm fractions was verified by Western blot analysis using β-actin as a cytosolic marker and COXIV as a mitochondrial marker (Figure 4B). Carotenoids were extracted from each fraction, and their molar amounts were quantified using HPLC with authentic standards (Figure 4C–F). Zeaxanthin was detectable in the total cell lysate and the two cellular fractions in both cell types. ARPE-19 (−) cells exhibited higher overall levels of zeaxanthin in total lysate and in the cytoplasm fraction when compared to ARPE-19 (+) cells. However, ARPE-19 (+) cells showed a six-fold increase in the mitochondrial zeaxanthin concentration when normalized to protein content (Figure 4D). This Aster-B-dependent enrichment of zeaxanthin in mitochondria was further confirmed by calculating the ratio of mitochondrial carotenoid relative to total cellular concentration (Figure 4F). These findings indicated that Aster-B facilitated lipid transport in ARPE-19 cells as observed by the transport of zeaxanthin from PM to mitochondria.

### 3.5. Oxidative Stress Modulates Carotenoid Transport Dependent on Aster-B

We next determined the effect of zeaxanthin on the survival of ARPE-19 cells under oxidative stress induced by hydrogen peroxide. For this purpose, ARPE-19 (+) cells and control ARPE-19 (−) cells were subjected to zeaxanthin uptake assays as described above (Figure 4A). Upon loading cells with zeaxanthin, they were subjected to H_2_O_2_ treatment for 24 h. Additional control groups in this experiment included ARPE-19 (+) and ARPE-19 (−) cells with no treatment, H_2_O_2_ alone, and supplementation with zeaxanthin without H_2_O_2_ exposure.

**Figure 5:**
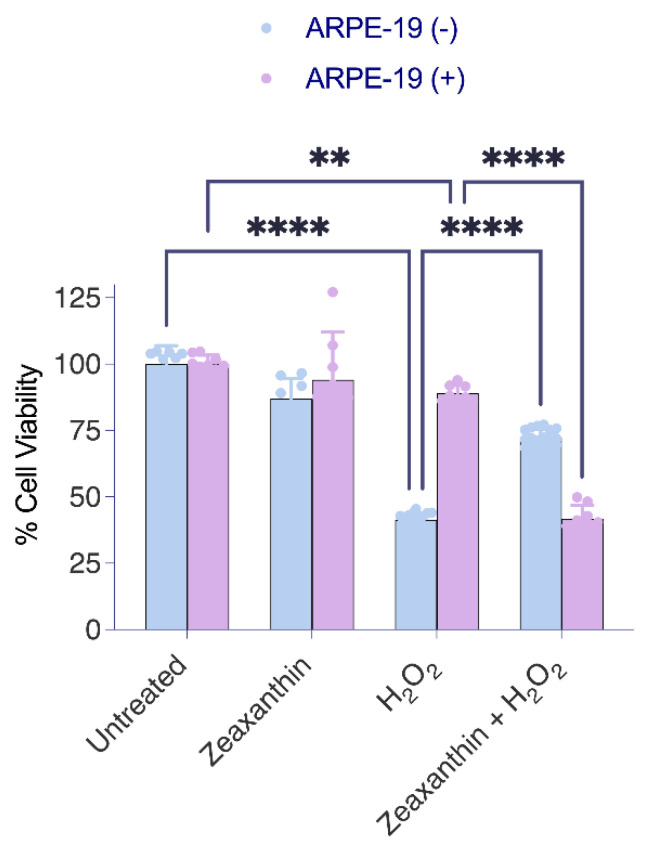
Aster-B expression modifies the protective function of zeaxanthin in H_2_O_2_-induced cell death. MTT assay in H_2_O_2_-treated ARPE-19 (+) cells showed significantly higher viability (87%) than H_2_O_2_-treated ARPE-19 (−) cells (52%). Zeaxanthin pretreatment significantly lowered the cell viability of H_2_O_2_-treated ARPE-19 (+) cells from 87% to 44%. Data shown are the mean ± SD of 4 to 6 independent experiments. Analysis was performed using one-way ANOVA using GraphPad Prism 10.2 software, and results are considered significant at ** *p* < 0.005, and **** *p* < 0.0001.

To assess the effects of the different treatment regimens on the viability of the cells, we used the MTT assay again (Figure 5). The analysis confirmed that ARPE-19 (+) cells showed higher resistance to ROS, showing greater survival rates than ARPE-19 (−) cells when exposed to H_2_O_2_ alone (Figure 5). The treatment with zeaxanthin alone had no significant effect on cell survival rates in either Aster-B-expressing or control ARPE-19 cells. In a combined treatment with zeaxanthin and H_2_O_2_, ARPE-19 (−) cells showed improved survival rates when compared to ARPE-19 (−) cells in the absence of zeaxanthin. Conversely, in ARPE-19 (+) cells, the presence of peroxide and zeaxanthin together resulted in decreased survival rates compared to peroxide treatment alone.

The outcome of this experiment indicated that Aster-B expression conferred increased resistance to oxidative stress in ARPE-19 cells. However, the presence of carotenoids modified this protective effect. While carotenoids enhanced survival in ARPE-19 (−) cells under oxidative stress, they reduced the survival rates of ARPE-19 (+) cells that transported carotenoids to mitochondria. These findings suggested that the effects of zeaxanthin under oxidative stress are dependent on Aster-B and on its subcellular localization.

### 3.6. Mitochondrial Carotenoids Are Oxidized in the Presence of Hydrogen Peroxide

Next, we analyzed whether hydrogen peroxide affected carotenoid transport in ARPE-19 (+) and ARPE-19 (−) cells. Thus, we grew cells and treated them with both zeaxanthin and H_2_O_2_. Upon the treatment procedure, cells were harvested and sub-fractioned into different cellular fractions. The purity of the fractions was verified by Western blot analysis using β-actin as a total cell lysate and COXIV as a mitochondrial marker (Figure 4B). Carotenoids were then extracted from each subcellular fraction and quantified using HPLC with an authentic zeaxanthin standard as control.

**Figure 6:**
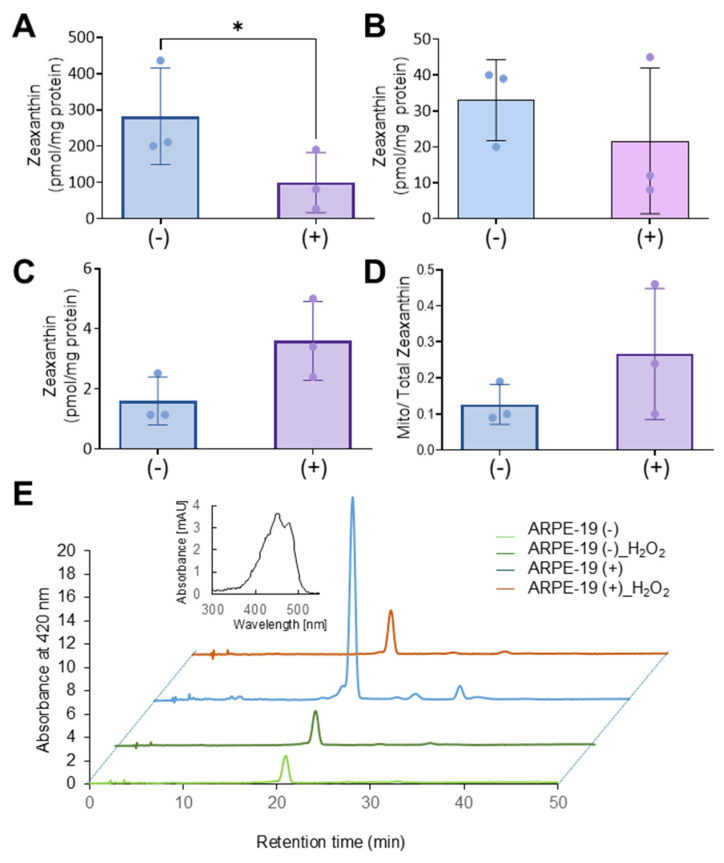
Oxidative stress affects the Aster-B-dependent accumulation of zeaxanthin in mitochondria. (**A**) Zeaxanthin concentration of total cell lysate, (**B**) mitochondria, (**C**) cytosol, and (**D**) the ratio of mitochondrial to cytoplasm zeaxanthin concentrations in ARPE-19 (+) and ARPE-19 (−) cells treated with 0.25 mM H_2_O_2_. (**E**) Representative HPLC traces at 420 nm of the mitochondrial fraction of ARPE-19 (+) and ARPE-19 (−) cells loaded with zeaxanthin and treated or not treated with H_2_O_2_. The major peak represents all-trans-zeaxanthin. The spectral characteristics of zeaxanthin are given in the inset. Data shown are the mean ± SD from three experiments per cell type and condition. Analysis was performed using an unpaired two-tailed *t* test using GraphPad Prism 10.2 software, and results are considered significant at * *p* < 0.05.

Zeaxanthin became detectable in all cellular fractions of both cell types (Figure 6A–D). ARPE-19 (−) cells exhibited significantly higher levels of zeaxanthin in total cell lysates when compared to ARPE-19 (+) cells. ARPE-19 (+) cells showed some enrichment of zeaxanthin in mitochondria when compared to ARPE-19 (−) cells. However, the enrichment of zeaxanthin in the mitochondrial fraction was far less pronounced than in ARPE-19 (+) cells in the absence of oxidative stress (compare Figure 6 with Figure 4).

We then investigated whether the reduced mitochondrial zeaxanthin concentration in ARPE-19 (+) was associated with the expression of β-carotene-oxygenase-2 (BCO2). BCO2 is a mitochondrial enzyme that converts zeaxanthin into apocarotenoids and has been shown to be upregulated by oxidative stress in liver carcinoma cells [23,24]. To explore this, we conducted qRT-PCR analysis, but BCO2 mRNA levels showed CT-values above 30, indicating that BCO2 was not expressed in ARPE-19 cells, even under oxidative stress conditions. Thus, we concluded that in conditions of oxidative stress, zeaxanthin was either not transported to mitochondria or rapidly degraded in ARPE-19 (+) cells.

## 4. Discussion

*GRAMD1B*, encoding the lipid carrier protein Aster-B, is emerging as a critical regulator of cholesterol homeostasis and isoprenoid lipid transport [11,12,17,25], including carotenoids [13,14]. This protein is expressed in the human neuronal retina and retinal pigment epithelium (RPE) [14], where lipid metabolism plays a pivotal role in cellular health [2]. Dysregulated lipid metabolism in the RPE, characterized by lipid-rich drusen accumulation and oxidative damage, is a hallmark of chronic ocular diseases such as age-related macular degeneration (AMD) [26]. To investigate *GRAMD1B*’s role, we developed an ARPE-19 cell model with CRISPR/dCas9-induced *GRAMD1B* expression and assessed its impact on lipid transport and oxidative stress responses.

Our findings provide evidence that Aster-B expression confers protective effects against oxidative stress, as ARPE-19 (+) cells expressing *GRAMD1B* exhibited reduced ROS levels and improved survival under hydrogen peroxide-induced stress. These effects appear to stem from Aster-B in maintaining membrane cholesterol homeostasis, highlighting its capacity to modulate oxidative stress independently of carotenoid transport. Additionally, lysosomes are essential for the degradation and recycling of lipid-rich photoreceptor outer segments. However, with aging or stress, lysosomal function can become compromised, leading to the accumulation of lipofuscin, an autofluorescent aggregate composed of oxidized lipids and proteins. Lipofuscin components, particularly bisretinoids such as A2E, have been shown to interfere with cholesterol metabolism by impairing cholesterol efflux from the lysosomal compartment, resulting in intracellular cholesterol accumulation [27]. This disruption in cholesterol homeostasis can contribute to lipid dysregulation, and Aster proteins may help to prevent this accumulation by moving cholesterol between membranes [28]. Cholesterol’s influence on cellular stress pathways, including the Nrf2 pathway and mitochondrial membrane integrity, aligns with our results [29].

By facilitating non-vesicular cholesterol transport [15], Aster-B likely supports cellular health, reducing ARPE-19 cells’ vulnerability to oxidative damage. Such a scenario is particularly relevant in AMD, where mitochondrial dysfunction and lipid dysregulation are key pathological features [5].

We used a commercial TF panel to detect oxidative stress pathways that are modulated by Aster-B. We observed a putative interplay between Aster-B and the tumor suppressor protein p53. It has been reported that cholesterol impacts p53 activity through mechanisms such as the oxysterol-mediated activation of apoptotic pathways and the modulation of membrane fluidity, which influence p53 localization and activity [30]. Aster-B’s role in cholesterol transport may indirectly affect p53-mediated stress responses, including oxidative stress and ER stress. Additionally, p53’s regulation of cholesterol metabolism genes, such as sterol regulatory element-binding proteins (SREBPs), may contribute to a potential feedback loop between Aster-B and p53 in maintaining lipid homeostasis and cellular health [31].

Nrf2 signaling was activated in both ARPE-19 cell lines. Interestingly, the Nrf2 target gene HO-1 was only detectable in ARPE-19 (−) but not ARPE-19 (+) cells (Figure 3 and Supplementary Figure S2). Aster-B expression likely modulates the oxidative stress response by altering intracellular lipid homeostasis and redox signaling, thereby impacting Nrf2-driven transcriptional activity in a complex manner. While Nrf2 protein levels are elevated in ARPE-19 (+) cells, the efficient transcriptional activation of downstream targets like HO-1 may require not only Nrf2 nuclear accumulation but also a favorable chromatin environment, intact redox-sensitive co-factors, and appropriate cellular context. Aster-B-mediated alterations in lipid and cholesterol distribution during oxidative stress could disrupt these regulatory mechanisms, preventing HO-1 induction despite increased Nrf2 levels. More research is needed to elucidate the biochemical and molecular details of this interaction.

Carotenoids, including lutein and zeaxanthin, add complexity to the oxidative stress response in human eyes. Known for their antioxidant properties, these pigments filter blue light and neutralize ROS, offering protection to ocular tissues [32,33,34,35]. Our study showed that Aster-B facilitates the intracellular movement of carotenoids in ARPE-19 cells. Interestingly, Aster-B expression had a pronounced effect on the response to hydrogen peroxide treatment. In ARPE-19 (+) cells, carotenoids surprisingly exacerbate the response to oxidative stress, as indicated by reduced survival rates as compared to control cells. By contrast, carotenoid treatment improved survival in ARPE-19 (−) cells when compared to controls. Such improved resistance to abiotic stress has been previously reported in carotenoid-treated ARPE-19 cells and might be related to the physical properties of the pigments [22,36]. The observations in ARPE-19 (+) cells suggest that excessive carotenoid accumulation in mitochondria may aggravate oxidative stress and apoptosis, revealing a potential trade-off in their protective roles. Interestingly, the adverse effects of carotenoids in ARPE-19 (+) cells in the presence of hydrogen peroxide were associated with reduced zeaxanthin levels in cells. The determination of BCO2 mRNA levels, encoding a carotenoid catabolizing enzyme, ruled out that this reduction went along with enzymatic carotenoid turnover. Therefore, we assume that the unspecific oxidation of carotenoids contributed to the reduced zeaxanthin levels in ARPE-19 (+) cells and the increased cell mortality in our experiments.

We and others previously have shown that a mitochondrial accumulation of carotenoids can be associated with cell death in the absence of BCO2 [23,37,38,39]. Notably, the RPE and many cell types of the neuronal retina express mitochondrial BCO2 [40,41,42], likely to protect them from detrimental carotenoid accumulation in mitochondria. Thus, our findings suggest that carotenoids act context- and location-dependently and provide either protection against ROS or exacerbate stress responses.

## 5. Conclusions

Our investigation highlights Aster-B’s structural and functional versatility. By forming contact sites between the PM, endoplasmic reticulum, and mitochondria, the lipid carrier facilitates the movement of lipids across cellular membranes, integrating cholesterol and carotenoid metabolism with stress responses. This structural organization positions Aster-B as a central player in maintaining cellular lipid balance and mitigating oxidative stress.

The clinical relevance of these findings is underscored by the success of carotenoid-based interventions, such as the Age-Related Eye Disease Study (AREDS), which demonstrated the protective effects of lutein and zeaxanthin against AMD progression [9,10]. Elevated high-density lipoprotein cholesterol levels and an altered composition in AMD patients further suggest that Aster-B’s role in cholesterol transport could influence disease outcomes [43]. Understanding how Aster proteins integrate with cholesterol-mediated pathways, such as NF-κB and Nrf2, and its interactions with carotenoids, could pave the way for targeted therapeutic strategies.

Therefore, future research should focus on delineating the mechanisms by which Aster-B coordinates cholesterol and carotenoid metabolism under stress conditions. Investigating its regulatory interactions with p53 and cholesterol-mediated signaling pathways, such as NF-κB and Nrf2, could provide deeper insights into its role in oxidative stress defense. Furthermore, optimizing the balance between carotenoids’ antioxidant properties and their potential pro-oxidant effects in an Aster-B-dependent context may improve therapeutic interventions. These efforts could significantly enhance our understanding of lipid dysregulation and oxidative stress in retinal diseases like AMD, offering promising avenues for disease management and treatment.

## Data Availability

The data supporting the findings of this study are contained within the article and the Supplementary Information. The raw data are available upon request from the corresponding author.

## Supplementary Materials

The following supporting information can be downloaded at: https://www.mdpi.com/article/10.3390/antiox14050575/s1, Figure S1: Generation of ARPE-19 (+) cells; Figure S2: Effects of Aster B expression on HO-1 protein expression.

## Abbreviations

The following abbreviations are used in this manuscript:

ARPE-19	Adult Retinal Pigment Epithelium 19
AMD	Age-related macular degeneration
H_2_O_2_	Hydrogen peroxide
SR-B1	Scavenger receptor class B type 1
HDL	High-density lipoprotein
SAM	Synergistic activation mediator
MTT	(3-(4,5-dimethylthiazol-2-yl)-2,5-diphenyltetrazolium bromide)
DCFH-DA	2′,7′-dichlorodihydrofluorescein diacetate
NFAT	Nuclear factor of activated T cells
ATF4	Activating transcription factor 4
AP-1	Activator protein 1
NRF2	Nuclear factor erythroid 2-related factor 2
HO-1	Heme-oxygenase-1
MCD	Methyl-β-cyclodextrin
BCO2	β-carotene-oxygenase-2
SREBPs	Sterol regulatory element-binding proteins,
AREDS	Age-Related Eye Disease Study
SMAD	Sma- and Mad-related proteins
NF-κB	Nuclear Factor kappa-light-chain-enhancer of activated B cells
STAT3	Signal Transducer and Activator of Transcription 3
