# Supplementary material for: Aster-B Modulates Oxidative Stress Responses and Carotenoid Distribution in ARPE-19 Cells
Source: Antioxidants (Basel). 2025 May 10;14(5):575. doi: 10.3390/antiox14050575 (PMC12108295; doi:10.3390/antiox14050575)
Supplement: Supplementary file 1 [file antioxidants-14-00575-s001.zip › antioxidants-3597117-supplementary.pdf]

antioxidants

MDPI

Supplementary Figure S1

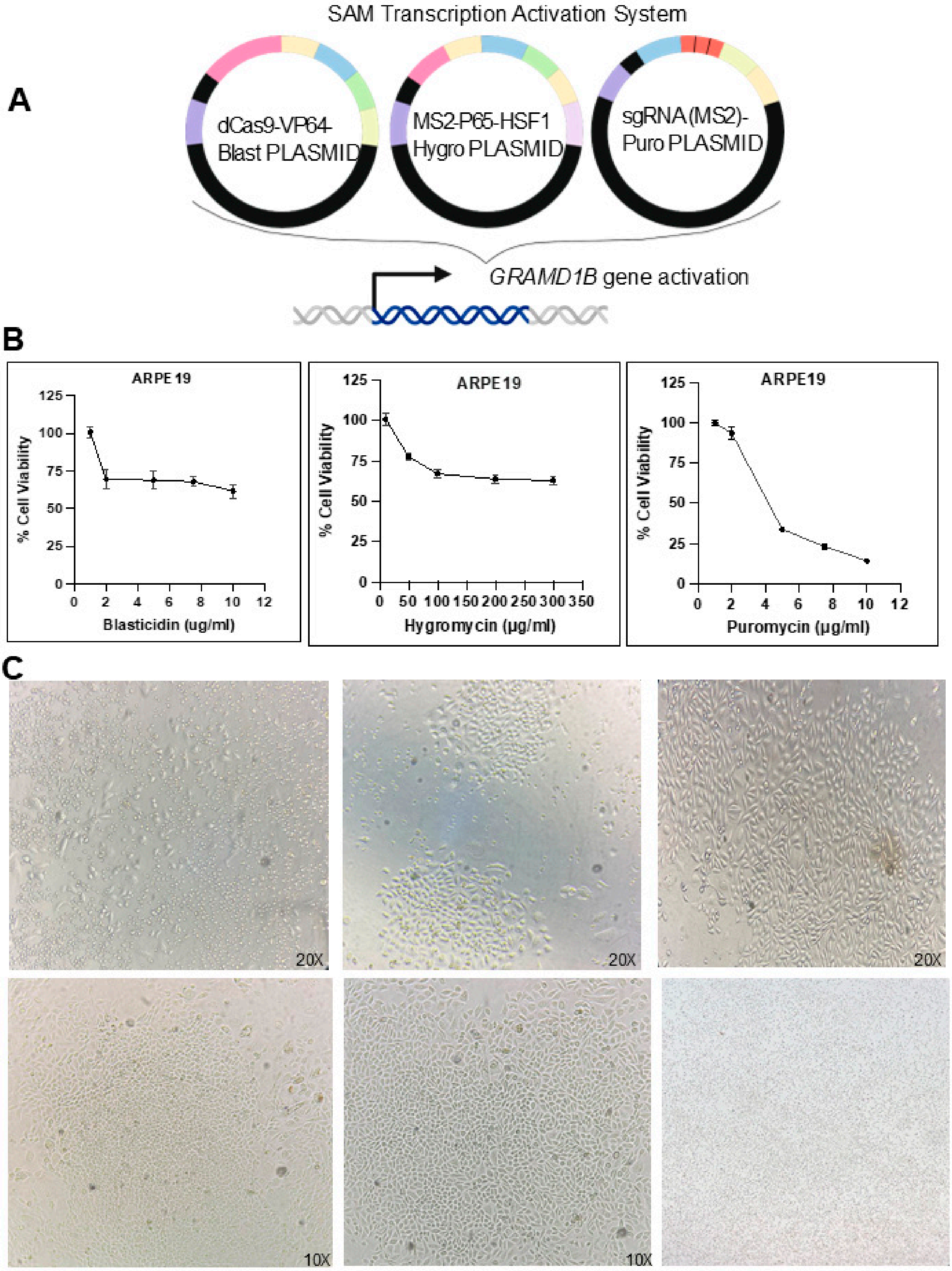

Supplementary Figure S1: Generation of ARPE-19 (+) cells: (A) The plasmid maps of *GRAMD1B* CRISPR Activation plasmid (h2). The plasmid maps are drawn using Bio-Render. It consists of the CRISPR/dCas9-VP64-Blast plasmid encoding the deactivated Cas9 (dCas9) nuclease (D10A and N863A) fused to the transactivation domain VP64, and a blasticidin resistance gene; the MS2-P65-HSF1-Hygro plasmid encoding the MS2-p65-HSF1 fusion protein, and a hygromycin resistance gene; the sgRNA (MS2)-Puro plasmid encoding a *GRAMD1B*-specific 20nt guide RNA, and a puromycin resistance gene. This synergistic activation mediator (SAM) transcription activation system binds to a specific site located upstream of the transcriptional start site (TSS) of the *GRAMD1B* gene and recruits transcription factors, thereby activating endogenous transcription of the *GRAMD1B* gene. **(B)** Viability curve for *GRAMD1B* null cells cultured in presence of varying concentration of Hygromycin, Blasticidin and Puromycin containing medium for 24 h. **(C)** Transfection with *GRAMD1B* plasmid DNA followed by selection and expansion of *GRAMD1B* stable cells. Null cells are transfected with 1.5 µg scrambled control or *GRAMD1B* plasmid DNA. Stable *GRAMD1B* cells are selected on medium containing Hygromycin, Blasticidin and Puromycin antibiotics for five days and stable colonies are further expanded on normal medium for one week.

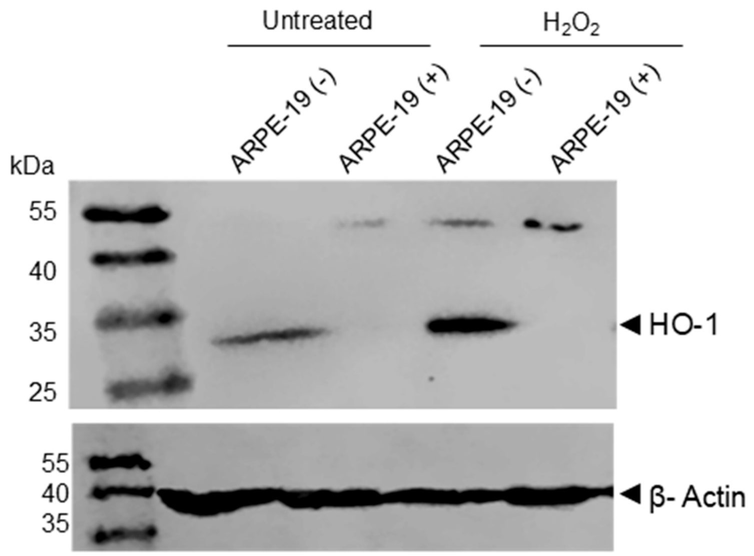

Supplementary Figure S2: Effects of Aster B expression on HO-1 protein expression. Representative Western blot of ARPE-19 (-) and ARPE-19 (+) untreated and 0.25 mM of H_2O_2 treated for 24 h cell lysates probed with anti-HO-1 and β-actin as a loading control. HO-1 expression was detected in ARPE-19 (-) cells but not in ARPE-19 (+) cells.
